# The Relation Between Fine Motor Skills and Executive Function in Two-Year-Old Children

**DOI:** 10.3390/bs16081262

**Published:** 2026-07-23

**Authors:** Lucas J. Rooney, Laura J. Claxton

**Affiliations:** Department of Health and Kinesiology, Purdue University, West Lafayette, IN 47907, USA; lucasjrooney@gmail.com

**Keywords:** fine motor skills, visual motor integration, executive function, toddlers

## Abstract

Cognitive processes such as executive function are associated with positive behavioral and health outcomes across the lifespan, highlighting the importance of identifying early predictors of executive functioning. During infancy and early childhood, the development of motor skills provides opportunities for action, exploration, and learning that may support emerging cognitive processes. The present exploratory study examined the relation between fine motor skills and executive function in 2-year-old children (*N* = 33; 13 girls and 20 boys; *M* = 31 months; age range = 25–35 months). Participants completed the fine motor subtests of the Peabody Developmental Motor Scales, assessing grasping and visual motor integration, and the Minnesota Executive Function Scale as a measure of executive functioning. Fine motor skills were positively associated with executive functioning; however, when controlling for age, only visual motor integration remained significantly correlated with executive functioning. A multiple linear regression analysis revealed that both grasping and visual motor integration significantly predicted executive functioning after controlling for age. These findings suggest that fine motor skills, especially the coordination of visual and manual processes, may play an important role in early executive function development independent of age. Interventions that support fine motor development may therefore have the potential to confer broader benefits for executive functioning in toddlers.

## 1. Introduction

Cognitive processes such as executive function are associated with a wide range of positive behavioral, academic, and health outcomes across the lifespan. Executive function supports goal-directed behavior by enabling individuals to coordinate thoughts and actions, and comprises three interrelated components: working memory, cognitive flexibility, and inhibitory control (Miyake & Friedman, 2012; Zelazo et al., 2016). Given the enduring importance of executive function for later functioning, identifying early predictors of executive function development is critical for informing prevention and intervention efforts.

From an embodied cognition perspective, emerging motor skills in infancy and early childhood play a central role in cognitive development (Thelen, 2000; Gottwald et al., 2016). This framework posits that cognition is grounded in action: as infants acquire new motor abilities, such as reaching, sitting, crawling, and walking, they gain novel opportunities to interact with and explore their environments. These motor-driven interactions support learning by enabling infants to generate goal-directed actions, plan motor sequences, and adapt behavior based on environmental feedback (e.g., Claxton et al., 2003; Gottwald et al., 2016). Such processes may be particularly relevant for the emergence of executive function, which itself is inherently goal-directed. Accordingly, infancy and early childhood represent a crucial developmental window for examining links between motor processes and executive function.

Executive function skills in early life have been shown to predict a broad array of outcomes later in development. Strong executive function abilities are associated with academic success, including higher performance in mathematics, reading comprehension, vocabulary, and general knowledge (Allan et al., 2014; Jacob & Parkinson, 2015). In contrast, poorer executive function in early childhood has been linked to less favorable health and behavioral outcomes, such as higher body mass index and eating-related concerns in later childhood (Graziano et al., 2013), as well as poorer health, increased criminal behavior, and suboptimal financial decision-making in adulthood (Moffitt et al., 2011). These findings underscore the importance of identifying early developmental correlates of executive function.

One candidate correlate that has received increasing attention is motor skill development. A growing body of research demonstrates associations between motor skills and executive function across development, particularly in typically developing children (McClelland & Cameron, 2019; Gandotra et al., 2022). Most empirical work in this area has focused on children between 3 and 12 years of age, with studies of preschool- and school-aged children consistently documenting positive relations between motor competence and executive function (e.g., Becker et al., 2014; Cameron et al., 2012; MacDonald et al., 2016; Oberer et al., 2017). A recent meta-analysis further demonstrated that balance ability and manual dexterity are among the strongest motor predictors of executive function in children aged 3 to 12 years (Gandotra et al., 2022). Notably, the strength of motor–cognitive associations appears to decrease with age, with younger children showing more robust relations between motor skills and executive function (van der Fels et al., 2015).

Motor skills are commonly categorized as either fine or gross motor skills. Fine motor skills involve the coordination of small muscle groups, primarily in the hands and fingers, and support activities such as grasping objects, drawing, and manipulating tools. In contrast, gross motor skills rely on larger muscle groups and include activities such as maintaining balance, locomotion, and whole-body coordination (Folio & Fewell, 2000). Although both domains contribute to development, fine motor skills may be particularly relevant for executive function because they often require precise coordination, visual guidance, and planning.

Despite evidence for stable individual differences in executive function emerging before 3 years of age (Devine et al., 2019; Hendry & Holmboe, 2021; Holmboe et al., 2018), relatively few studies have examined relations between motor skills and executive function in children under 3 years of age. Existing findings in this age range are mixed. Gottwald et al. (2016) reported no concurrent associations between fine or gross motor skills and executive function in 18-month-old infants. In contrast, Wu et al. (2017) found that fine and gross motor skills assessed at 2 years predicted executive function at 3 years. Importantly, although Wu et al. (2017) demonstrated longitudinal associations, they did not address whether motor skills and executive function are concurrently related at 2 years of age. Thus, there is limited evidence regarding concurrent relations between motor skills and executive function in toddlers.

The present study focused specifically on fine motor skills for three primary reasons. First, fine motor skills have been shown to predict executive function and academic outcomes more consistently than gross motor skills in early childhood (Cameron et al., 2012; van der Fels et al., 2015). Second, individual differences in reaching and grasping kinematics at 2 years of age are predictive of stable individual differences in fine motor performance at 3 years (Chen et al., 2010), suggesting that meaningful variability in fine motor skills is already present by this age. To our knowledge, comparable evidence for stable individual differences in gross motor skills in neurotypical toddlers is limited. Third, there remains a notable gap in the literature examining fine motor skills and executive function concurrently in children younger than 3 years of age.

In the current study, fine motor skills were assessed using experimenter-administered subtests of the Peabody Developmental Motor Scales, Second Edition (PDMS-2; Folio & Fewell, 2000), which measure grasping and visual motor integration. Grasping assesses the child’s ability to use the hands to manipulate objects, whereas visual motor integration assesses the integration of visual perceptual skills with fine motor control during tasks such as copying shapes. Executive function was assessed using the Minnesota Executive Function Scale (MEFS; Carlson & Zelazo, 2014), an age-appropriate, tablet-based card-sorting task that captures cognitive flexibility, working memory, and inhibitory control. Parents also completed a demographic questionnaire.

Based on prior evidence linking fine motor skills and executive function (e.g., Gandotra et al., 2022; van der Fels et al., 2015), it was predicted that fine motor skills, including grasping and visual motor integration, would be positively associated with executive functioning at 2 years of age. Specifically, children with stronger fine motor skills were expected to demonstrate higher executive function performance.

## 2. Methods

### 2.1. Participants

Thirty-three 2-year-olds (13 girls, Mean Age = 31 months of age, Range = 25- to 35-months) participated in the study. Participants were part of a larger study assessing cognitive and motor development in early childhood. Participants were eligible if they were between 24 and 35 months of age and able to complete the study procedures in English. Exclusion criteria included documented developmental delays affecting task completion and failure to complete at least two of the three study tasks. Therefore, 4 additional toddlers were tested but excluded from analysis because of an inability to understand English (*n* = 1), a documented language delay (*n* = 1), and not completing at least 2 out of the 3 tasks (*n* = 2). Socioeconomic status was indexed via parental education level. Families were generally of high socioeconomic status, with 77% of mothers and 81% of fathers holding at least a bachelor’s degree. Families were predominately White (82%) with smaller proportions identifying as Asian (6%), Black (3%), Hispanic (3%), or undisclosed (6%). Families were recruited using a convenience sampling approach through community outreach efforts, including daycare centers, flyers, local events, and public records. Interested families contacted the research team and were screened for eligibility prior to enrollment. Testing took place at the Purdue University Motor Development Laboratory or at two Purdue University affiliated childcare sites. Children received a small toy (value of approximately $5) for participating. The Purdue University Institutional Review Board approved all procedures, and informed consent was obtained from the caregivers prior to participation.

### 2.2. Procedures

Children completed the Minnesota Executive Function Scale (Version 1.3) and the fine motor subtests of the Peabody Developmental Motor Scale Version-2. Assessments were administered by a trained experimenter following a standardized task order to minimize variability across participants. The Minnesota Executive Function Scale was administered first, followed by the fine motor subtests of the Peabody Developmental Motor Scale. Caregivers were given a demographic questionnaire to complete during the laboratory visit or via email prior to the testing session.

#### 2.2.1. Executive Function Measure

Minnesota Executive Function Scale Version 1.3 (Carlson & Zelazo, 2014). Executive functioning was assessed using the Minnesota Executive Function Scale, a tablet-based application administered individually by an experimenter. The Minnesota Executive Function Scale is validated for children between 2 and 13 years of age and is designed to assess executive function as a unified construct encompassing working memory, cognitive flexibility, and inhibitory control. Children begin the task at an age-appropriate level determined by the application.

For each level of the task, children use specific rules to sort virtual cards into one of two boxes (e.g., matching cards by animal type). For example, children are told to place the cow cards in the cow box and then the cat cards in the cat box. As children progress through the scale, the difficulty level increases such that children also need to switch the rules that they are following. For example, children may first sort the cards to match size (e.g., the big elephant cards go in the big elephant box and the little elephant cards go in the little elephant box). Children then apply a second, contradictory rule or play a “silly game” where they now sort according to the opposite size (e.g., this time the big elephant cards will go in the little elephant box and the little elephant cards will go in the big elephant box.)

The Minnesota Executive Function Scale is a comprehensive measure of executive function as all three components of executive function are taxed throughout the assessment (Carlson & Zelazo, 2014). Children utilize working memory to hold the rule in mind and use the current rule state to sort the given card. Children must inhibit the desire to sort the current card based on the picture only, instead following the current rule state. Finally, flexibly shifting from one rule to the next challenges the child’s cognitive flexibility. However, the Minnesota Executive Function Scale is not designed to look at these individual aspects of executive function, but executive function ability as combination of its constructs. The Minnesota Executive Function Scale is highly correlated with other full-length batteries of executive function (Carlson & Zelazo, 2014).

The scale is also highly adaptive in that it starts at an age-dependent level and then increases in difficulty. Children must be correct on at least four out of five trials to continue to the next level. If the child fails at the predetermined appropriate age-based starting level, the program automatically provides an easier level until the child’s current level of executive function is reached. It takes approximately three to six minutes to administer. Performance data are automatically uploaded to the Reflection Sciences platform. For 2-year-olds, the outcome scores are based on accuracy (the number of cards correctly sorted by the child). Higher numbers of correctly sorted cards equated to better executive function performance (Carlson & Zelazo, 2014). Three children did not complete the Minnesota Executive Function Scale and were excluded from analyses involving executive function.

#### 2.2.2. Fine Motor Control Measures

Peabody Developmental Motor Scale-2nd Edition (Folio & Fewell, 2000). Fine motor skills were assessed using the Grasping and Visual Motor Integration subtests of the Peabody Developmental Motor Scale-2nd edition, a standardized assessment of motor development for children from birth to 72 months of age. Administration and scoring followed procedures outlined in the Examiner’s Manual.

The Grasping subtest assesses a child’s ability to control hand and finger movements, including tasks such as grasping objects, manipulating blocks, using a marker, and fastening buttons. The Visual Motor Integration subtest evaluates the coordination of visual perception and fine motor control through tasks such as copying shapes, folding paper, stacking blocks into forms, and threading beads. Testing for each subtest began at the age-appropriate starting point corresponding to items that 75% of children in the normative sample are expected to pass.

Each task was scored on a 3-point scale (0–2), with higher scores indicating greater mastery. For example, in a Grasping task requiring children to unbutton a strip of three buttons, a score of 2 was assigned if all buttons were unfastened within 75 s, a score of 1 if completed in more than 75 s, and a score of 0 if the child was unable to complete the task. For a Visual Motor Integration task involving folding a piece of paper, a child received a score of 2 for producing a clear crease, a score of 1 for crumpling the paper, and a score of 0 for merely touching the paper.

Children were permitted up to three attempts per item. Consistent with standardized testing procedures, testing continued until both basal and ceiling levels were established for each subtest. A basal level was defined by mastery of three consecutive items, and a ceiling level was reached when a child received a score of 0 on three consecutive items. If a child became fatigued or temporarily unwilling to complete an item, testing proceeded to the next item and returned to incomplete items later in the subtest when possible.

Raw subtest scores for Grasping and Visual Motor Integration were calculated by summing points earned up to the ceiling item, assigning full credit (2 points) for items below the basal level. Age-normed scores were not used in the present analyses. One child did not complete the Grasping subtest and one child did not complete the Visual Motor Integration subtest.

#### 2.2.3. Data Analysis

SPSS (Version 29.0.2.0) was used for all analyses. A MANOVA was conducted to examine the effect of sex on age, executive function (Minnesota Executive Function Scale scores), and fine motor skills (Grasping, Visual Motor Integration). The multivariate effect of sex was not statistically significant, Pillai’s Trace = 0.133, *F*(4, 23) = 0.884, *p* = 0.49, partial η^2^ = 0.133; therefore, sex was collapsed across all analyses.

Pearson correlation coefficients were computed to examine the bivariate relationships among age (in months), executive function, and fine motor skills. To further explore the unique contributions of fine motor skills to executive functioning independent of age, partial correlations were conducted controlling for age (in months). All correlation analyses were conducted using pairwise deletion to maximize the use of available data.

To further examine whether fine motor skills uniquely predicted executive functioning while controlling for age, a multiple linear regression analysis was performed. The Minnesota Executive Function Scale scores served as the dependent variable, with Grasping and Visual Motor Integration entered as predictors. Age in months was included as a covariate to account for developmental differences. Prior to analysis, the data were screened for missing values, and cases with incomplete data on any of the relevant variables were excluded using listwise deletion. All assumptions of linear regression were assessed and met prior to analysis.

## 3. Results

Descriptive statistics for all study variables, including means, standard errors, and ranges, are presented in Table 1.

### 3.1. Correlations

**Figure 1:**
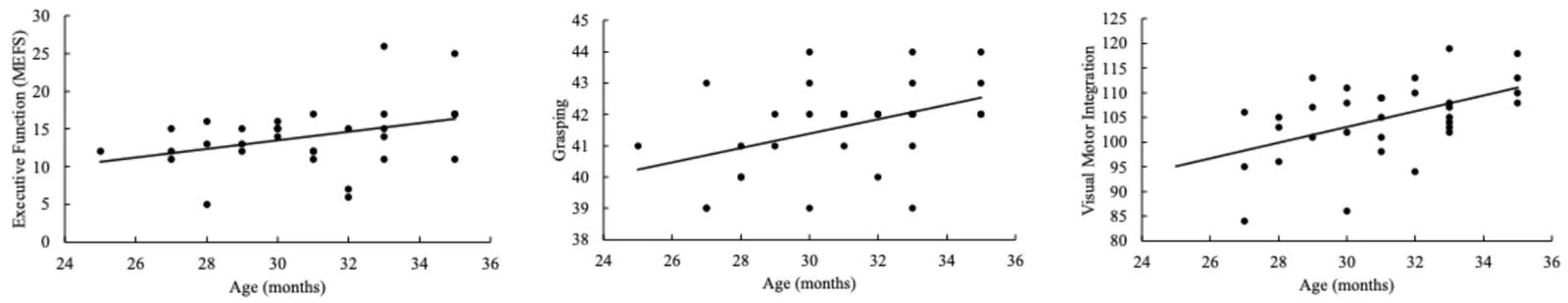
Scatterplots depicting relationships between age in months and executive functioning (Minnesota Executive Function Scale), Grasping, and Visual Motor Integration scores. Solid lines represent linear regression fits.

Scatterplots depicting relationships between age (in months), executive functioning (Minnesota Executive Function Scale) and fine motor skills are shown in Figure 1. Pearson correlations revealed significant moderate positive correlations between age and Visual Motor Integration (*r* = 0.50, *p* = 0.004) and Grasping (*r* = 0.43, *p* = 0.015). In contrast, the association between age in months and the Minnesota Executive Function Scale was not statistically significant (*r* = 0.34, *p* = 0.063).

**Figure 2:**
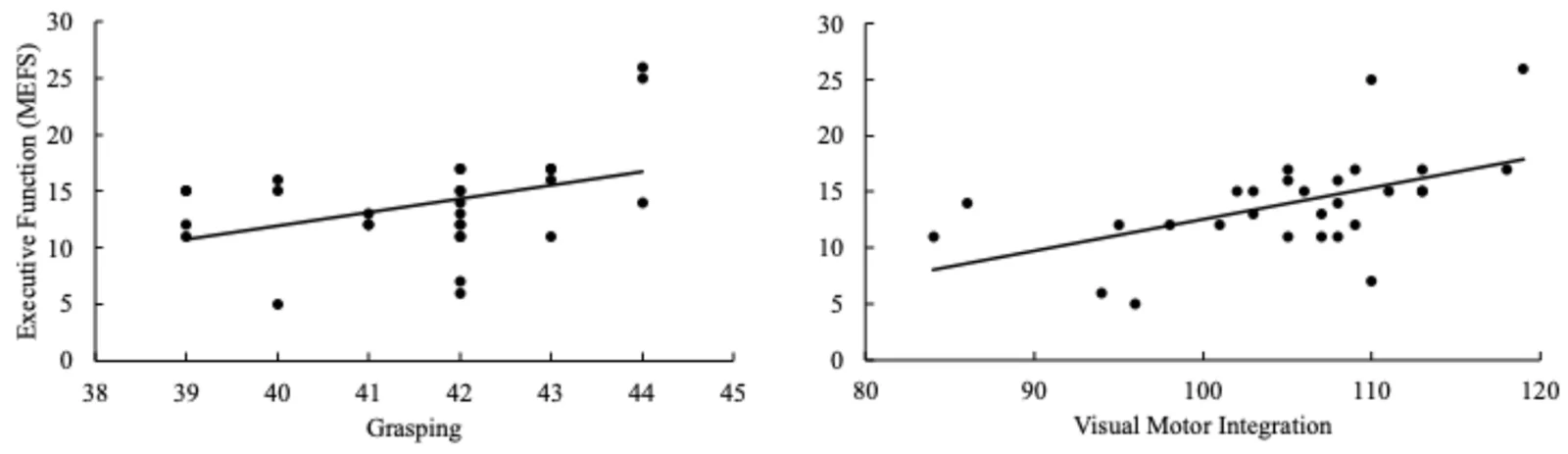
Scatterplots depicting relationships between executive functioning (Minnesota Executive Function Scale) and fine motor skills (Grasping and Visual Motor Integration) scores. Solid lines represent linear regression fits.

Scatterplots illustrating relations between executive functioning and each fine motor skill are shown in Figure 2. Executive functioning was significantly positively correlated with Grasping (*r* = 0.40, *p* = 0.031) and Visual Motor Integration (*r* = 0.52, *p* = 0.004). Grasping and Visual Motor Integration did not correlate with each other (*r* = 0.09, *p* = 0.615). However, given that age was positively associated with both fine motor skills and executive functioning, the observed associations between executive functioning and fine motor skills may reflect, in part, shared developmental variance resulting from concurrent developmental change.

To further explore the relation between fine motor skills and executive functioning independent of age, partial correlations controlling for age in months were conducted. After controlling for age, executive functioning and Visual Motor Integration remained positively correlated (*r* = 0.43, *p* = 0.023). In contrast, the correlation between executive functioning and Grasping was no longer significant (*r* = 0.30, *p* = 0.122).

### 3.2. Multiple Linear Regression Analysis

To determine whether fine motor skills uniquely predicted executive functioning beyond age-related effects, a multiple linear regression was conducted with Grasping and Visual Motor Integration entered as predictors and age in months included as a covariate (Table 2). The overall regression model was statistically significant, *F*(3, 24) = 5.57, *p* < 0.01, and explained 41% of the variance in executive functioning scores (*R*^2^ = 0.41).

Both Grasping (*B* = 1.26, *p* = 0.028) and Visual Motor Integration (*B* = 0.32, *p* = 0.007) were significant predictors of executive functioning, while age was not a significant covariate (*B* = -0.30, *p* = 0.448).

## 4. Discussion

The present study explored whether fine motor skills, specifically grasping and visual motor integration, predict executive functioning in 2-year-old children. As predicted, fine motor skills were positively associated with executive functioning. Although both Grasping and Visual Motor Integration significantly predicted executive functioning in the regression model, Visual Motor Integration emerged as the stronger and more consistent predictor across analytic approaches. In contrast, the association between grasping and executive function was less consistent, suggesting that grasping may explain unique variance in executive functioning when considered alongside other fine motor skills, but may be more sensitive to age-related developmental influences.

Importantly, the observed associations between executive functioning and fine motor skills must be interpreted in the context of developmental age. Age was positively associated with both visual motor integration and grasping and showed a positive, although non-significant, association with executive functioning. Thus, some of the age-uncorrected associations likely reflect shared developmental progress across domains. Consistent with this interpretation, when controlling for age, only visual motor integration remained significantly associated with executive functioning. This pattern highlights the importance of accounting for age-related variance when examining motor–cognitive relations during early childhood and suggests that visual motor integration may represent a more age-independent correlate of executive functioning than grasping.

### 4.1. The Role of Visual Motor Integration in Executive Function

Whereas both fine motor skills were related to executive functioning, the stronger association between visual motor integration and executive functioning suggests that tasks requiring coordinated visual-motor control are closely tied to cognitive control Fmechanisms. One possible explanation for this finding relates to differences in the nature of the tasks included in the two Peabody Development Motor Scales subtests. Grasping tasks primarily emphasized how a child executed a motor action (e.g., whether the child used a pincer grasp), whereas Visual Motor Integration tasks emphasized the outcome of goal-directed actions (e.g., the successful construction of a block structure or accurate copying of shapes). Visual Motor Integration tasks also required the coordination of multiple motor components over time, potentially increasing cognitive demands relative to grasping tasks.

Previous research indicates that task complexity can influence the strength of associations between motor skills and executive functioning, with more complex motor tasks showing stronger relations to executive function (van der Fels et al., 2015). Tasks such as building towers or manipulating tools may require the simultaneous engagement of working memory to maintain task goals, inhibitory control to suppress ineffective actions, and cognitive flexibility to adjust strategies as needed. However, this explanation alone may be insufficient, as Maurer and Roebers (2019) found that both simple and complex fine motor tasks were related to executive function. Thus, complexity may contribute to—but does not fully explain—the observed pattern.

An alternative interpretation is that the integration of visual and motor information itself represents a shared process underpinning performance in both visual motor and executive function tasks. Numerous studies have emphasized the importance of visual–motor coordination for successful performance across motor and cognitive domains (Cameron et al., 2016; Maurer & Roebers, 2019). From this perspective, visual motor integration may reflect underlying attentional and control processes that are also central to executive functioning. Supporting this view, MacDonald et al. (2016) found that visual motor integration skills in preschool-aged children predicted later executive functioning, whereas object manipulation skills—conceptually similar to grasping—were more strongly associated with social behaviors than executive function. A similar pattern may already be present by two years of age.

### 4.2. Measurement Considerations

Another potential explanation for the differential strength of observed relationships concerns the sensitivity of the fine motor measures used. Although the Peabody Developmental Motor Scale is a widely used standardized assessment, it may not optimally capture individual differences in fine motor skills among two-year-old children. In the present sample, the Grasping subtest exhibited relatively low variability, likely reflecting ceiling or floor effects for specific tasks. For example, tasks requiring unbuttoning a strip of buttons appeared to exceed the motor capabilities of most children in this age group, resulting in uniformly low performance. In contrast, the Visual Motor Integration subtest produced greater variability overall, although certain tasks (e.g., scissor use) also appeared developmentally misaligned for some children, potentially due to limited prior experience rather than motor ability per se.

These findings highlight the limitations of relying solely on standardized outcome-based measures to assess fine motor development in very young children. Future research may benefit from the use of more fine-grained, process-oriented measures—such as kinematic analyses of reaching, grasping, or object manipulation—to better capture meaningful variability in fine motor control (Chen et al., 2010). Such approaches may allow for more precise identification of which aspects of motor behavior are most closely linked to executive functioning during early development.

### 4.3. Limitations

Several limitations should be considered when interpreting the present findings. First, the relatively small sample size limits statistical power and generalizability. Given the exploratory nature of the study and the challenges associated with recruiting and testing toddlers, an a priori power analysis was not conducted. Consequently, the present findings should be interpreted cautiously until replicated in larger samples. Although visual motor integration showed a robust association with executive functioning across analyses, a larger sample may clarify whether grasping has a more modest or context-dependent relation with executive function at this age. In addition, because age was associated with both fine motor performance and executive functioning, disentangling shared developmental change from domain-specific associations remains challenging during this period of rapid development. Although age-controlled analyses helped address this concern, future studies with larger samples and broader age ranges may provide a clearer understanding of these relations.

Second, the correlational design precludes conclusions regarding causality or developmental directionality. Although the findings are consistent with developmental cascade accounts, whereby motor development supports emerging executive functioning, alternative explanations cannot be ruled out. For instance, Diamond (2000) proposed that motor and executive processes share overlapping neural substrates, such that observed associations reflect common underlying brain mechanisms rather than causal influences between domains.

Third, the sample was relatively homogeneous with respect to socioeconomic status and ethnicity. As a result, the findings may not generalize to populations with different demographic characteristics, and future research should include more diverse samples.

### 4.4. Implications for Intervention and Future Research

Despite these limitations, the present findings have important implications for early intervention. Motor-based interventions in preschool-aged children have been shown to improve executive functioning (Mulvey et al., 2018), and two years of age may represent a particularly sensitive developmental period in which fine motor experiences can support cognitive control. During this period, foundational motor skills involving reaching, grasping, and object manipulation—such as role-differentiated bimanual manipulation—are rapidly developing (Needham & Nelson, 2023; Nelson et al., 2025; Thompson et al., 2025). From an embodied cognition perspective, providing children with enriched opportunities to engage in visually guided motor activities may promote executive function development by strengthening the coordination between perception, action, and cognitive control processes (Gibson, 1988; Thelen, 2000).

Future research should examine these relations longitudinally to determine how early fine motor skills relate to later executive functioning over time. Longitudinal designs will be particularly important for clarifying developmental pathways linking motor and executive function development. Future studies should also include more diverse samples to improve generalizability and incorporate both fine and gross motor measures to better understand their relative contributions to executive functioning. It may also be valuable to investigate potential mediating mechanisms, such as attention, self-regulation, or visual-motor coordination, that help explain motor–cognitive associations. Additionally, future work should explore how different types of fine motor tasks relate to specific components of executive function and experimentally evaluate whether interventions targeting visual motor integration produce measurable improvements in executive functioning during early childhood.

More broadly, the present findings highlight the need for additional research examining motor–cognitive relations during the toddler period. Although substantial research has documented associations between motor skills and executive functioning in preschool- and school-aged children, comparatively fewer studies have examined these relations in toddlers despite rapid developmental changes occurring during this period. One challenge is the difficulty of obtaining developmentally sensitive measures that capture meaningful individual differences during this period. Future research would benefit from incorporating more process-oriented approaches, such as kinematic analyses, eye-tracking methods, wearable sensors, and longitudinal designs capable of capturing developmental change at a finer timescale. Extending this work to children at elevated developmental risk, including those with motor delays or other neurodevelopmental concerns, may also help clarify whether visual motor integration serves as an early behavioral marker of later executive functioning outcomes. Addressing these methodological challenges may be particularly important for reducing the current gap in developmental research focused on the toddler years. Such efforts would contribute to a more comprehensive understanding of developmental pathways during the toddler years and help address the broader gaps identified within toddler-focused developmental science.

## 5. Conclusions

Overall, these results highlight the importance of fine motor development, especially visual motor integration, as an important correlate of executive functioning in 2-year-old children. These results contribute to a growing body of evidence demonstrating close links between motor and cognitive development in early childhood and suggest that visually guided fine motor skills may represent promising targets for early identification and intervention efforts aimed at supporting executive function development in typically developing children.

## Figures and Tables

**Table 1:** Descriptive Statistics for Age, Executive Function, and Fine Motor Tasks.

Variable	n	M	SE	Range
Age (mo)	33	30.88	0.46	25.00–35.00
Executive Function Task				
MEFS	30	13.90	0.81	5.00–26.00
Fine Motor Task				
Grasping	32	41.59	0.25	39.00–44.00
VMI	32	104.78	1.39	84.00–119.00

Note. MEFS = Minnesota Executive Function Scale; VMI = Visual Motor Integration.

**Table 2:** Multiple linear regression predicting executive functioning from fine motor skills and age.

Predictor	*B*	SE	*t*	*p*
Intercept	−62.87	21.67	−2.90	0.008
Age (months)	−0.30	0.39	−0.77	0.448
Grasping	1.26	0.54	2.33	0.028
Visual Motor Integration	0.32	0.11	2.96	0.007

Note**.** *B* = unstandardized regression coefficient; SE = standard error. Age is measured in months. The overall model was significant, *F*(3, 24) = 5.57, *p* < 0.01, *R*^2^ = 0.41.

## Data Availability

The raw data supporting the conclusions of this article will be made available by the authors upon request.

## References

[B1-behavsci-16-01262] Allan N. P., Hume L. E., Allan D. M., Farrington A. L., Lonigan C. J. (2014). Relations between inhibitory control and the development of academic skills in preschool and kindergarten: A meta-analysis. Developmental Psychology.

[B2-behavsci-16-01262] Becker D. R., Miao A., Duncan R., McClelland M. M. (2014). Behavioral self-regulation and executive function both predict visuomotor skills and early academic achievement. Early Childhood Research Quarterly.

[B3-behavsci-16-01262] Cameron C. E., Brock L. L., Murrah W. M., Bell L. H., Worzalla S. L., Grissmer D., Morrison F. J. (2012). Fine motor skills and executive function both contribute to kindergarten achievement. Child Development.

[B4-behavsci-16-01262] Cameron C. E., Cottone E. A., Murrah W. M., Grissmer D. W. (2016). How are motor skills linked to children’s school performance and academic achievement?. Child Development Perspectives.

[B5-behavsci-16-01262] Carlson S. M., Zelazo P. D. (2014). Minnesota executive function scale: Test manual.

[B6-behavsci-16-01262] Chen Y. P., Keen R., Rosander K., Von Hofsten C. (2010). Movement planning reflects skill level and age changes in toddlers. Child Development.

[B7-behavsci-16-01262] Claxton L. J., Keen R., McCarty M. E. (2003). Evidence of motor planning in infant reaching behavior. Psychological Science.

[B8-behavsci-16-01262] Devine R. T., Ribner A., Hughes C. (2019). Measuring and predicting individual differences in executive functions at 14 months: A longitudinal study. Child Development.

[B9-behavsci-16-01262] Diamond A. (2000). Close interrelation of motor development and cognitive development and of the cerebellum and prefrontal cortex. Child Development.

[B10-behavsci-16-01262] Folio M., Fewell R. F. (2000). Peabody developmental motor scales.

[B11-behavsci-16-01262] Gandotra A., Csaba S., Sattar Y., Cserényi V., Bizonics R., Cserjesi R., Kotyuk E. (2022). A meta-analysis of the relationship between motor skills and executive functions in typically-developing children. Journal of Cognition and Development.

[B12-behavsci-16-01262] Gibson E. J. (1988). Exploratory behavior in the development of perceiving, acting, and the acquiring of knowledge. Annual Review of Psychology.

[B13-behavsci-16-01262] Gottwald J. M., Achermann S., Marciszko C., Lindskog M., Gredebäck G. (2016). An embodied account of early executive-function development: Prospective motor control in infancy is related to inhibition and working memory. Psychological Science.

[B14-behavsci-16-01262] Graziano P. A., Kelleher R., Calkins S. D., Keane S. P., Brien M. O. (2013). Predicting weight outcomes in preadolescence: The role of toddlers’ self-regulation skills and the temperament dimension of pleasure. International Journal of Obesity.

[B15-behavsci-16-01262] Hendry A., Holmboe K. (2021). Development and validation of the early executive functions questionnaire: A carer-administered measure of executive functions suitable for 9-to 30-month-olds. Infancy.

[B16-behavsci-16-01262] Holmboe K., Bonneville-Roussy A., Csibra G., Johnson M. H. (2018). Longitudinal development of attention and inhibitory control during the first year of life. Developmental Science.

[B17-behavsci-16-01262] Jacob R., Parkinson J. (2015). The potential for school-based interventions that target executive function to improve academic achievement: A review. Review of Educational Research.

[B18-behavsci-16-01262] MacDonald M., Lipscomb S., McClelland M. M., Duncan R., Becker D., Anderson K., Kile M. (2016). Relations of preschoolers’ visual-motor and object manipulation skills with executive function and social behavior. Research Quarterly for Exercise and Sport.

[B19-behavsci-16-01262] Maurer M. N., Roebers C. M. (2019). Towards a better understanding of the association between motor skills and executive functions in 5-to 6-year-olds: The impact of motor task difficulty. Human Movement Science.

[B20-behavsci-16-01262] McClelland M. M., Cameron C. E. (2019). Developing together: The role of executive function and motor skills in children’s early academic lives. Early Childhood Research Quarterly.

[B21-behavsci-16-01262] Miyake A., Friedman N. P. (2012). The nature and organization of individual differences in executive functions: Four general conclusions. Current Directions in Psychological Science.

[B22-behavsci-16-01262] Moffitt T. E., Arseneault L., Belsky D., Dickson N., Hancox R. J., Harrington H., Houts R., Poulton R., Roberts B. W., Ross S., Sears M. R., Thomson W. M., Caspi A. (2011). A gradient of childhood self-control predicts health, wealth, and public safety. Proceedings of the National Academy of Sciences of the United States of America.

[B23-behavsci-16-01262] Mulvey K. L., Taunton S., Pennell A., Brian A. (2018). Head, toes, knees, SKIP! Improving preschool children’s executive function through a motor competence intervention. Journal of Sport and Exercise Psychology.

[B24-behavsci-16-01262] Needham A. W., Nelson E. L. (2023). How babies use their hands to learn about objects: Exploration, reach-to-grasp, manipulation, and tool use. Wiley Interdisciplinary Reviews: Cognitive Science.

[B25-behavsci-16-01262] Nelson E. L., Danforth C., Needham A. W. (2025). Infant reaching and grasping: Frameworks for testing developmental cascades. Infant Behavior and Development.

[B26-behavsci-16-01262] Oberer N., Gashaj V., Roebers C. M. (2017). Motor skills in kindergarten: Internal structure, cognitive correlates and relationships to background variables. Human Movement Science.

[B27-behavsci-16-01262] Thelen E. (2000). Motor development as foundation and future of developmental psychology. International Journal of Behavioral Development.

[B28-behavsci-16-01262] Thompson P. A., Arnold A. J., Ambike S., Claxton L. J. (2025). Role differentiated bimanual manipulation during a lab-based free play task. Infant Behavior and Development.

[B29-behavsci-16-01262] van der Fels I. M., Te Wierike S. C., Hartman E., Elferink-Gemser M. T., Smith J., Visscher C. (2015). The relationship between motor skills and cognitive skills in 4–16 year old typically developing children: A systematic review. Journal of Science and Medicine in Sport.

[B30-behavsci-16-01262] Wu M., Liang X., Lu S., Wang Z. (2017). Infant motor and cognitive abilities and subsequent executive function. Infant Behavior and Development.

[B31-behavsci-16-01262] Zelazo P. D., Blair C. B., Willoughby M. T. (2016). Executive function: Implications for education. NCER 2017-2000.

